# Effects of dietary sodium on performance, flight and compensation strategies in the cotton bollworm, *Helicoverpa armigera *(Hübner) (Lepidoptera: Noctuidae)

**DOI:** 10.1186/1742-9994-7-11

**Published:** 2010-04-13

**Authors:** Kai Xiao, Ke Shen, Jian-Feng Zhong, Guo-Qing Li

**Affiliations:** 1 Department of Entomology, Nanjing Agricultural University; Key Laboratory of Monitoring and Management of Plant Diseases and Pests, Ministry of Agriculture, Nanjing, 210095, China

## Abstract

**Background:**

Sodium is critical for many physiological functions in insects. Herbivorous insects should expend considerable energy to compensate for sodium deficiency due to low sodium concentration in most inland plants upon which they feed. However, sodium compensation behaviors such as mud-puddling have been observed in some species but not in others. We expect that there may be other sodium compensation strategies in insects. Here, we select a rarely mud-puddling insect species, the cotton boll worm, *Helicoverpa armigera*, and determine the effects of dietary sodium on performance and flight, and examine their means of sodium compensation.

**Results:**

When freshly hatched *H. armigera *neonates were cultured on one of three diets differing in sodium contents (diet A, B and C with a high, middle and low sodium concentrations, respectively), the larvae on diet C grew larger, had a higher mortality rate and a shorter development period than those on diet A and B. The larvae previously fed from 1^st ^to 3^rd ^instar on diet C consumed more subsequent diet when they were transferred to diet A or C at 4^th ^instar, comparing to those previously fed on diet A. Moreover, any 4^th^-instar larvae on diet C consumed a greater amount of food than those on diet A, no matter which diet the larvae had previously ingested from 1^st ^to 3^rd ^instar. Moths from diet A and B flew more rapidly than those from diet C, with similar sugar and lipid utilization rates among the three test groups. When a 5^th^-instar cannibal from diet A, B or C and a 5^th^-instar victim from diet A were housed together, many more cannibals from diet C ate their victims. When a victim from diet A, B or C was provided, a cannibal from diet C was more likely to eat the victim from diet A. When newly emerged moths had been exposed to 3% sodium chloride solution for all scotophase period, the average weight increase (proxy for sodium solution intake) for moths from diet A was lower than those from diet B or C.

**Conclusion:**

Sodium-deficient diet resulted in rapid growth and development of *H. armigera *larvae, decreased larvae survival, and reduced flight speed of *H. armigera *adults. To compensate for sodium deficiency, *H. armigera *ingested a large quantity of larval food, increased larval cannibalism incidence and harvested sodium during the adult stage.

## Background

Sodium plays important roles in osmotic balance, in neuromuscular system, and in digestion and excretion process in animals [1-4]. Therefore, animals have a dietary sodium requirement [5-7]. Moreover, body sodium is tightly regulated in some animals [6,8].

Sodium concentration is very low in most inland plants [9-12]. In contrast, the herbivores that eat them maintain much higher sodium levels. In vertebrates, sodium concentration was 100- to 1000-fold more than that in plants [3]. In insects, the sodium concentrations in the haemolymph of phytophages in Orthoptera, Phasmida and Lepidoptera were much higher than that in plants. Moreover, the adult haemolymph of most holometabolous insects had higher sodium concentrations than that of the larvae. In the Lepidopterans *Mamestra brassicae *and *Bombyx mori*, for example, the sodium concentration in the adult blood was 3 and 8 times more than that in the larvae blood, respectively [13]. It seems that herbivores must expend considerable energy to find and harvest sodium [3,4,14-16].

Many vertebrates (cattle, chamois, cow, elk, deer, goat, gorillas, kangaroos, moose, parrots, rabbit, sheep, and stags) have been documented to exhibit an appetite for salt in nature [9,15,17-19]. In invertebrates, some butterflies [20-30], moths [21,31-33], honeybees [34], ants [3], cicadellids [35] and *Ceracris kiangsu *adults [4] searched for potential salt sources such as moist ground, perspiration, tears, excrements, or rotten fish. This phenomenon was defined as mud-puddling and some of these insect species, such as the notodontid moth, *Gluphisia septentrionis*, have been proven to mud-puddle to collect sodium [12].

Interestingly, mud-puddling behaviors were often observed in some insect species but rarely or never found in others. If sodium deficiencies are common in herbivores [10,36-40], one can expect that non-puddling insect herbivores have other sodium compensation strategies.

Arms et al. [1] and Downes [21] suggested that sodium might be important for maintaining high neuromuscular activity. In Riodinidae, 124 species in 41 genera were collected and male feeding behavior and morphology relationship was analyzed [25]. Among these species, fish carrion feeders and puddlers had lower mean wing area to thoracic volume ratios (WA:TV) than flower nectarers. Hall and Willmott [25] found that WA:TV ratio was significantly negatively correlated with flight speed and oxygen consumption (a direct indicator of metabolic rate), and suggested that fish carrion feeders and puddlers may obtain some substances to provide the necessary nutrients to maintain high metabolic rates during rapid flight. Proving the sodium content in fish (0.6 mg/g) is high, this result indicates that sodium is the most possible candidate for fish carrion feeders and puddlers in Riodinidae to maintain rapid flight. The results reported by Molleman et al. [29], however, did not support the indication. They found sodium concentration in the species with the smallest WA:TV ratio was relatively low [29]. Therefore, we need further research to confirm the effect of sodium on flight speed and flight duration.

The cotton bollworm, *Helicoverpa armigera *(Hübner) (Lepidoptera: Noctuidae) continues to challenge crop production throughout much of its distribution in tropical and subtropical region of the Old World [41,42]. The adults are hardly found to mud-puddle. Here, we varied the sodium concentrations in a *H. armigera *larval artificial diet to obtain resulting larvae, pupa and adults and addressed the following questions. (1) How does dietary sodium influence larvae performance and adult flight? and (2) How does *H. armigera *compensate for sodium deficiency?

## Methods

### General methods

*H. armigera *larvae were collected from cotton plants (*Gossypium hirsutum*) at Nanjing (32.0N, 118.5E), Jiangsu Province in China in July and August 2008 and routinely reared in an insectary under controlled temperature (28 ± 1°C), photoperiod (14 h light/10 h dark) and relative humidity (70-80%) until pupation. Females and males were then segregated based on the morphology of the abdominal terminal segments of the pupa. Newly emerged moths were given unlimited access to a 10% sucrose solution, and allowed to mate for two successive nights. Eggs were collected, sterilized and transferred to a 1000 ml glass pot according to the method of Xu et al. [43] and Liu et al. [44]. Between 12 and 24 h prior to egg hatching, an artificial diet was offered. The diet contained wheat-germ powder (27.5 g), soybean flour (7.5 g), yeast (2.0 g), agar (2.5 g), water (93 ml), Wesson salt mixture (0. 2 g) [45], vitamins (vitamin C 0.7 g, B1 0.41 mg, B2 0.82 mg, B6 0.41 mg, B12 0.01 mg, biotin 0.04 mg; nicotinamide 1.63 mg, folic acid 0.41 mg; calcium pantothenate 1.63 mg), inositol (32.64 mg) and citric acid (0.2 g). When larvae developed to the late second instar, they were transferred individually to a 15 ml glass vial containing a piece of the artificial diet until pupation.

To estimate the effects of dietary sodium on performance, flight and compensation strategies in *H. armigera *larvae or adults, three larval diets differing in sodium concentrations were prepared. Diet A was a conventional diet. Diet B was modified from the conventional diet in which half amounts of sodium chloride (NaCl) and sodium fluoride (NaF) in Wesson salt mixture were replaced with equal mole of potassium chloride (KCl) and potassium fluoride (KF), respectively. Diet C was modified from the conventional diet in which all NaCl and NaF in Wesson salt mixture were replaced with equal mole of KCl and KF. After dried and ashed in a muffle furnace, the concentrations of sodium and potassium in the three diets were determined by an atomic absorption spectrophotometer. The average concentration of sodium in diet A, B and C is 0.43 ± 0.013, 0.30 ± 0.010 and 0.14 ± 0.005 mg/g dry weight (n = 5), and that of potassium is 3.12 ± 0.094, 3.47 ± 0.134, and 3.78 ± 0.143 mg/g dry weight (n = 5), respectively. Between 12 and 24 h prior to egg hatching, one of the three diets was offered. The resulting larvae, pupa or adults were used in experiments.

Observations of growth parameters were made for the larvae that were cultured on the three different diets. Survival rate from 1^st ^to 3^rd ^instar, body weight increase, development duration, the rate of larvae to reach the pupal stage (pupation rate), pupa weight, emergence rate, adult weight, and percent of moth with abnormal wing shape were determined. For each test diet 30 to 64 individuals were observed and repeated three times.

To determine the effect of previous food intake on subsequent food consumption by larvae, the larvae previously fed on diet A from 1^st ^to 3^rd ^instar, and then the resulting 4^th^-instar larvae were transferred to diet A or C, respectively. Likewise, those previously fed on diet C from 1^st ^to 3^rd ^instar were also split into two groups, one was provided with fresh diet C, and the other with diet A. The diet weight was measured before transfer and 24 h after the transfer. Since our preliminary measure showed that the artificial diet in a 15 ml glass vial lost little water over a 24 h period, the consumption amount of subsequent food by each larva was calculated as the difference between the two weights.

To test sodium harvest by adults, one-day-old moths originated from diet A, B or C were introduced individually into a 100 ml jar, given unlimited access to a 3% sodium solution during scotophase, a 10 h dark phase from 8:00 PM to 6:00 AM. The body weights of the moths were weighed before and after scotophase, and the average weight increase was proxy for sodium solution intake.

### Chemicals

Sodium chloride (NaCl), sodium fluoride (NaF), potassium chloride (KCl), potassium fluoride (KF), potassium iodide (KI), potassium dihydrogen phosphate (KH_2_PO_4_), alum (KAl(SO_4_)_2_·12H_2_O), calcium phosphate (Ca_3_(PO_4_)_2_), calcium carbonate (CaCO_3_), ferric phosphate (FePO_4_), magnesium sulfate (MgSO_4_), cupric sulfate (CuSO_4_), and manganese sulfate (MnSO_4_) were purchased from Shantou Xilong Chemical Factory and were mixed of the composition in Wesson [45] to make Wesson salt mixture. Vitamin C, B1, B2, B6, B12, biotin, nicotinamide, folic acid, calcium pantothenate, inositol, and citric acid were obtained from Sigma (St. Louis, MO). Anthrone (C_14_H_10_O), sulfuric acid (H_2_SO_4_), and trichloroacetic acid (C_2_HCl_3_O_2_) were from Sinopharm Chemical Reagent Co. Ltd. Ethanol (C_2_H_5_OH), ethyl acetate (C_4_H_8_O_2_), glucose (C_6_H_12_O_6_), and ethyl ether (C_4_H_10_O) were from Shanghai Experiment Reagent Co. Ltd. All had claimed purities of 99%.

### Cannibalism test

Cannibalism was observed by a method similar to Simpson et al. [16]. The 5^th^-instar larvae fed on diet A, B or C were selected as cannibals or victims. The victims were freshly incapacitated by rupture of the head capsule to make them can not injure cannibals. A cannibal and a victim were housed in a culture dish (12 cm in diameter and 2 cm in height). Each treatment was replicated 20 to 30 times. A victim was classified as cannibalized if body parts were either partially or entirely missing. One hour later the cannibalism incidences were counted.

### Tethered flight test

All flight tests were done with a computer-interfaced tethered flight system similar to that of Naranjo [46] and Murata and Tojo [47]. Each one-day-old moth (24 h after emergence) was anesthetized under carbon dioxide (exposure time was about 1 min), and the scales on the back of thorax were removed using a small brush. The moth was then anesthetized under carbon dioxide and adhered to one end of a lightweight lever, which was balanced by a small flag attached to the opposite end and was pivoted on a fulcrum. After recovery from anesthesia, the moth was allowed to fly and the lever rotated around the fulcrum. The rotation cycles of the lever were recorded and the flight duration and distance for each moth over a 24-h dark phase were calculated by the computer.

### Total carbohydrates and lipid analyses

Before or after flight, the male and female moths were frozen and lyophilized at -20°C. The moths were weighed, and the total lipid was extracted with chloroform-methanol (2:1 v/v) according to Folch et al. [48] and total carbohydrates were extracted with boiling water. Total lipid was determined by the phospho-vanillin method as described by Ayali and Pener [49] and total carbohydrate was tested by the anthrone method [50].

### Statistical analysis

The data were given as means ± SE. Statistical tests included one-way, repeated measures and 2 × 2 factorial ANOVAs. A repeated measures ANOVA was used to test for effects of different diets and feeding days on larval growth. A 2 × 2 factorial ANOVA was used to test for effect of previous food intake on subsequent food consumption by larvae, with 1^st ^to 3^rd ^instar pre-treatment diet as the first factor and the subsequent diet as the second factor. The differences among treatments were compared by Duncan's New Multiple Range Test [51]. Differences in cannibalism incidences were compared by a chi-square test.

## Results

### Effects of diet on growth of the larvae

Larval body weights were affected by diets, feeding days and the interaction between them (Table 1). The mean body weight of the larvae fed on diet C was heavier than those on diet A or B after 7 days' culture. Statistically significant differences among them were observed after 10 days' culture and further increased from then on (Table 2).

**Table 1:** A repeated-measures ANOVA for effect of diet and larvae age (days after hatching) on larvae body weight

*Source*	*df*	*MS*	*F*	*P*
Diet	2	21818.20	8.61	0.0003
Error a	188	2535.45		
				
Larvae age	1.85	2957949.84	1167.02	<0.0001
Larvae age × Diet	3.71	12545.52	4.95	0.0010
Error b	348.40	2534.63		

Above statistical computations were performed by the program SPSS 16.0 for Windows (SPSS for Windows xp; SPSS, Inc., Chicago, IL). *H. armigera *larvae were fed on diet A, B and C. Diet A was a conventional diet. Diet B was modified from the conventional diet in which half amounts of NaCl and NaF in Wesson salt mixture were replaced with equal mole of KCl and KF, respectively. Diet C was modified from the conventional diet in which all NaCl and NaF in Wesson salt mixture were replaced with equal mole of KCl and KF.

**Table 2:** The mean body weights of the *H. armigera *larvae feeding on diet A, B and C

**Larvae fed on** **Diet**	Larvae body weight (mg) *				
	
	**L7**	**L8**	**L9**	**L10**	**L11**
A	21.1 ± 0.3	36.5 ± 1.2	85.8 ± 2.3	122.2 ± 5.4 d	211.5 ± 9.6 B
B	20.9 ± 0.4	35.7 ± 1.0	86.6 ± 2.0	134.6 ± 6.0 Cd	226.3 ± 7.2 B
C	21.8 ± 0.3	39.0 ± 1.2	98.4 ± 2.0	140.9 ± 5.0 C	257.9 ± 8.1 A

* Data are shown as Mean ± SE for 64 individuals. L7 to L11 represents 7 to 11 days after the larvae transferred to one of the three diets. Different capital or small letters after values indicate significant differences at *P *< 0.01 or at *P *< 0.05 among different treatments by a repeated measures ANOVA and Duncan's multiple range test (DMRT). See text and Table 1 for further explanations.

The larval survival rate from 1^st ^to 3^rd ^instar of the three test groups fed on diet A, B and C was 85.6 ± 1.7%, 70.4 ± 2.1%, and 65.6 ± 1.9% respectively, the former was significantly higher than the latter two. Development time of the three test groups on diet A, B and C was 14.9 ± 0.13 days, 14.8 ± 0.13 days and 14.4 ± 0.14 days, respectively, the former two developed significantly slower than the latter. The pupation rates (93.8 ± 8.4%, 91.7 ± 7.7% and 92.7 ± 8.3%, respectively) and pupa weights (302.7 ± 13.3 mg, 311.7 ± 9.3 mg and 298.9 ± 11.8 mg, respectively) of the three test groups on diet A, B and C showed little differences.

### Effects of previous diet intake on subsequent food consumption

**Figure 1:**
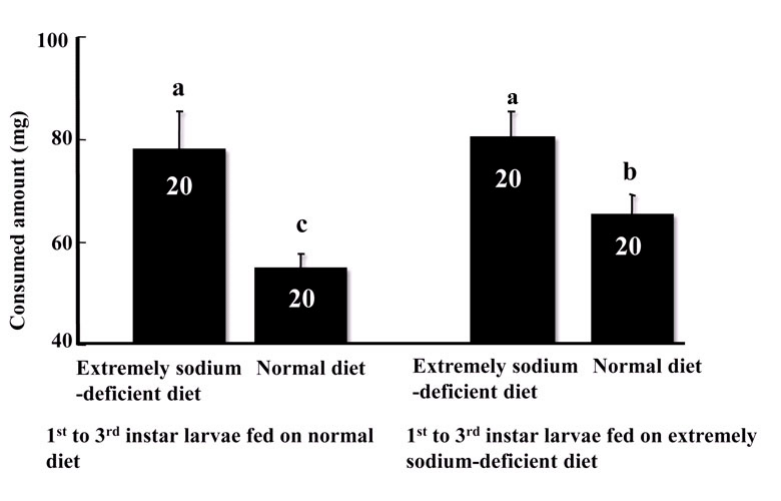
**Food consumption of 4^th^-instar larvae on diet A or C**. The larvae previously fed on diet C or A from 1^st ^to 3^rd ^instar, and then were transferred to diet A or C, respectively. Values represent the mean ± SEM and followed by the same small letter are not significantly different at *P *< 0.05 compared by a 2 × 2 factorial ANOVA and Duncan's New Multiple Range Test. Numbers of replicates are shown inside the bars.

The effect of previous food intake on subsequent food consumption by larvae was analyzed. The larvae previously fed from 1^st ^to 3^rd ^instar on diet C consumed more subsequent diet when they were transferred to diet A or C at 4^th ^instar, compared to those previously fed on diet A (*F*(1,92) = 4.65, *P *< 0.05). Moreover, any 4^th^-instar larvae on diet C consumed a greater amount of food than those on diet A, no matter which diet the larvae had previously ingested from 1^st ^to 3^rd ^instar (*F*(1,92) = 25.2, *P *< 0.01). The interaction effect of pre-treatment diet and the consumption of the subsequent diet was not significant (*F*(1,92) = 0.46, *P *> 0. 1) (Figure 1).

### Effects of larval diet on the adults

The emergence rates of the three test groups from diet A, B and C were similar. Moths from diet B and C had higher rates of abnormal wings. Moreover, the mean body weights of both males and females from diet B and C were lighter than those from diet A (Table 3).

**Table 3:** Influences of larval diets on the *H. armigera *adult emergence and body weight

	Larvae fed diet	Emergence percent*	Percent of moth with abnormal wing shape*	Body weight (mg)*
Male	A	83.5 ± 5.9 a	8.3 ± 0.6 b	141.4 ± 5.2 a
	B	86.2 ± 4.6 a	10.5 ± 0.8 b	133.9 ± 3.6 ab
	C	84.3 ± 6.1 a	14.5 ± 0.9 a	126.5 ± 4.0 b
Female	A	84.1 ± 8.7 a	9.5 ± 0.6 b	158.6 ± 3.1 a
	B	84.8 ± 5.7 a	11.1 ± 0.7 ab	139.2 ± 5.5 b
	C	84.9 ± 7.4 a	14.5 ± 1.2 a	138.0 ± 7.2 b

* Data are shown as Mean ± SE for at least 10 individuals. Different small letters after values indicate significant differences (*P *< 0.05) among different treatments by a one-way ANOVA and Duncan's multiple range test (DMRT). See text and Table 1 for further explanations.

### Effects of larval diets on flight capacity in the adults

Twenty four hours after emergence, flight capacities of the non-fed moths derived from the larvae that fed on diet A, B and C were tested using a tethered flight system. During a period of 24 h test, the total flight durations were, on an average, from 10 to 11 h, with those from diet A sustained for a slightly shorter period of time. Moreover, the flight speeds of the moths from diet A and B were significantly faster and the flight distances were a little longer than those from diet C (Table 4).

**Table 4:** Effects of larval diets on flight capacities in the adults

Larvae fed on diet	n	Flight speed (km.h^-1^) *	Flight duration (h) *	Flight distance (km) *
A	30	2.51 ± 0.04 a	9.96 ± 0.65 a	26.61 ± 1.35 a
B	28	2.44 ± 0.05 a	10.91 ± 0.40 a	26.01 ± 1.04 a
C	33	2.32 ± 0.05 b	10.82 ± 0.54 a	24.93 ± 1.38 a

* Data are shown as Mean ± SE. Different small letters after values indicate significant differences (*P *< 0.05) among different treatments by a one-way ANOVA and Duncan's multiple range test (DMRT). See text and Table 1 for further explanations.

### Effects of larval diets on flight fuel in the adults

The average sugar content per gram of dry body weight was highest in one-day-old non-fed moths from diet C, followed by that in moths from diet A and then that in moths from diet B. The amount of total lipid per gram of dry body weight was significantly highest in one-day-old non-fed moths from diet B among the three test groups (Table 5).

**Table 5:** Differences in total amounts of sugar and lipid (mg/g)in the moths from the larvae on diet A, B and C, and their variations before and after flight

Larvae fed diet	Sugar*			Lipid*		
	
	Before flight	After flight	Difference	Before flight	After flight	Difference
A	68.3 ± 4.5 b	46.6 ± 3.4 b	21.8 ± 1.5 a	405.8 ± 30.5 b	231.4 ± 26.8 a	174.4 ± 14.4 b
B	65.8 ± 4.9 ab	41.8 ± 2.4 b	24.0 ± 2.9 a	478.6 ± 15.6 a	255.7 ± 16.4 a	222.9 ± 12.1 a
C	78.0 ± 3.8 a	52.1 ± 4.0 a	26.2 ± 1.8 a	430.8 ± 29.8 ab	240.6 ± 18.6 a	190.3 ± 11.3 a

* Data are shown as Mean ± SE for 12-18 individuals. Different small letters after values indicate significant differences (*P *< 0.05) among different treatments by a one-way ANOVA and Duncan's multiple range test (DMRT). See text and Table 1 for further explanations.

After more than 10 h's flight, the sugar and lipid levels reduced. The sugar amounts in the moths from diet A, B and C decreased by 31.9%, 36.5% and 33.6%, respectively. Meanwhile, the lipid quantities of the three test groups from diet A, B and C decreased by 43%, 46.6% and 44.2% (Table 5).

### Effects of larval diets on larvae cannibalism

**Figure 2:**
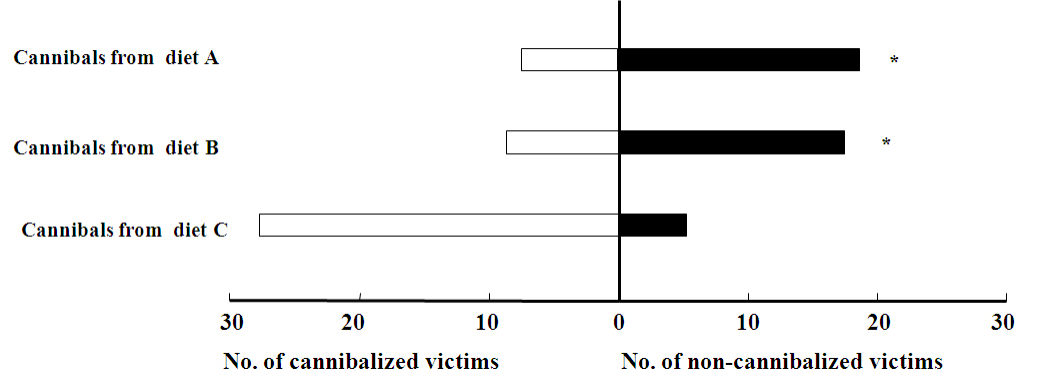
**State-dependent changes in cannibalism incidence**. A 5^th^-instar cannibal from diet A, B or C and a 5^th^-instar victim from diet A were housed together for 1 hour. * indicates statistically significant difference in cannibalism incidence between cannibals from diet C and those from diet A or B by a chi-square test.

**Figure 3:**
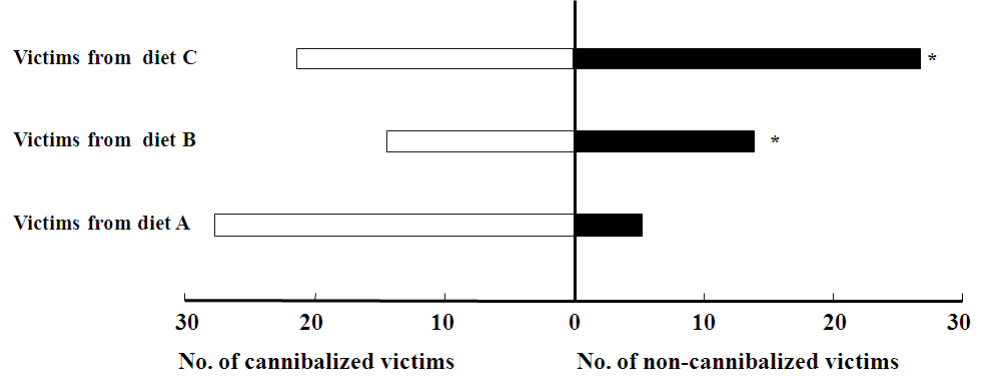
**Victim-dependent changes in cannibalism incidence**. A 5^th^-instar cannibal from diet C and a 5^th^-instar victim from diet A, B or C were housed together for 1 hour. * indicates statistically significant difference in cannibalism incidence between victims from diet A and those from diet B or C by a chi-square test.

When a 5^th^-instar victim from diet A and a 5^th^-instar cannibal from diet A, B or C were left together for 1 hour, much more cannibals from diet C ate their victims, comparing to the cannibals from diet A and B (Figure 2). When a 5^th^-instar victim from diet A, B or C was provided, respectively, a 5^th^-instar cannibal from diet C was more likely to eat the victim from diet A (Figure 3).

### Effects of larval diets on sodium intake by the adults

The sodium intake of the one-day-old moths was determined by comparing the mean body weight before and after 10 h of exposure to 3% sodium chloride solution. The average weight increase for 10 males from diet A, B and C was -2.7% ± 0.2%, 3.1% ± 0.6% and 10.7% ± 0.3%, respectively. The latter was significantly higher than the former two. Meanwhile, the weight increase for 10 females from diet A, B and C was -0.04% ± 0.1%, 10.8% ± 0.3% and 10.1% ± 0.4%, respectively. The latter two were significantly higher than the former (*F*(2,27) = 3.68, *P *= 0.039).

## Discussion

Exploring the influence of minerals in artificial diets has proven difficult because of impurities in crude natural materials such as wheat germ, soybean flour, yeast and agar, in chemical additives such as amino acids, sterols, vitamins, and fatty acids, and in antioxidants and antimicrobials [52]. To circumvent this difficulty, here we prepared three larval diets and measured their actual sodium and potassium concentrations. Diet A is a conventional diet. Since it has been applied successfully to culture *H. armigera *in our laboratory for more than twenty years, we assume that sodium content in diet A is suitable for *H. armigera *larvae. Diet B or C is modified from diet A by replacing a half or whole amount of sodium in Wesson salt mixture [45] with equal mole of potassium. The sodium concentration in diet B and C decreased by 30.2% and 67.4%, respectively, whereas the potassium concentration increased 11.2% and 21.2%, respectively. Since *H. armigera *is a phytophagous species and so presumably adapted to ingesting much higher proportion of potassium than characterize conventional mammalian salt mixtures, such as Wesson salt mixture [52], 10% potassium increase in diet B and 20% increase in diet C should have little effect on *H. armigera *larvae.

Our results showed *H. armigera *larvae on diet C grew larger, had a shorter (but not much) development period than those on diet A and B. The larvae previously fed from 1^st ^to 3^rd ^instar on diet C consumed more subsequent diet when they were transferred to diet A or C at 4^th ^instar, comparing to those previously fed on diet A. Moreover, any 4^th^-instar larvae on diet C consumed a greater amount of food than those on diet A, no matter which diet the larvae had previously ingested from 1^st ^to 3^rd ^instar. These results suggest that the caterpillars on sodium deficient diet practice a compensatory feeding response. Similar results have been reported in *Locusta migratoria *[53].

Moreover, compensatory feedings for sodium were also showed in other lepidopterans, especially in males. Male larvae of *Thymelicus lineola*, for example, consume about 85% of the foliage consumed by female larvae, yet on adult emergence males have a dry weight only 48% of that of the females. Consequently, at emergence males contain 82% of the total body sodium of females and the concentration is twice that in females [54]. This suggests that caterpillars, especially male caterpillars, exhibit compensatory feeding for sodium. Similarly, each notodontid moth *Gluphisia septentrionis *male contains about 2.3 μg of sodium and had an average body weight of 70 mg, the average sodium concentration in male was 32.9 μg/g fresh weight and approximately 329 μg/g dry weight, 110 times more than that in their primary larval host plant quaking aspen (*Populus tremuloides*) [12]. *Heliothis virescens *male larvae were found to have assimilated significantly more sodium from artificial diet than female larvae [55]. In some butterfly species such as *Kallimoides rumia, Bicyclus graueri*, *Euphaedra medon *and *E. alacris*, puddling is uncommon in both sexes, but males do transfer sodium to the female during copulation [29]. A great proportion of sodium in males should then be derived from larval feeding.

Sodium stress may also be tempered by feeding at higher trophic levels [16]. Mormon crickets, *Anabrus simplex*, showed a higher incidence of cannibalism within migratory bands, and NaCl prefeeding substantially reduced the incidence of cannibalism in laboratory test [16]. Cannibalism was also observed in *H. armigera *larvae, especially under poorer nutritional conditions or greater larval rearing density [56]. Is cannibalism a strategy for *H. armigera *larvae to obtain sodium? Our results showed that the larval survival rate from 1^st ^to 3^rd ^instar of the three test groups fed on diet A, B and C was 85.6 ± 1.7%, 70.4 ± 2.1%, and 65.6 ± 1.9% respectively, the former was significantly higher than the latter two. In cannibalism test, when a 5^th^-instar victim from diet A and a 5^th^-instar cannibal from diet A, B or C were left together for 1 hour, many more cannibals from diet C ate their victims. Furthermore, when a 5^th^-instar victim from diet A, B or C was provided, a 5^th^-instar cannibal preferred victims with putatively higher sodium concentrations. Our results demonstrated that *H. armigera *larvae frequently cannibalized others under sodium deficient condition.

Our results also showed that the moths from diet A obtained less average weight increase than those from B or C after having had the opportunity to feed on 3% sodium chloride solution for 10 h scotophase period. This demonstrated that sodium-deprived *H. armigera *adults could resort to puddling to compensate for the shortage of sodium. If considing the excrement, the differences among the resulting moths were much greater, since we noted the bottoms of the jars containing diet B or C originated moths were wetter than those containing diet A originated moths.

In present paper, we tested the flight capabilities of the moths from diet A, B and C. We found that the moths from diet A and B flew more rapidly and covered slightly longer distances than those from diet C, with similar sugar and lipid utilization rates among the three test groups. These findings may have two possible explanations, of which the deduction proposed by Downes [21] and Arms et al. [1] is one. They suggested that sodium may be important for maintaining high neuromuscular activity and may affect flight speed. The moths originating from sodium-deficient diets had putatively lower sodium concentrations in their body and flew slowly. The other explanation is size differences among the moths from three different diets. Our results showed that the moths from diet B and C had lighter body weights and consequently smaller body size, and tend to fly slower.

Larvae on diet C consume a large quantity of food, and grew larger than caterpillars on diet A. However, the resulting pupae and adults from diet C have lighter body weights than those from diet A. Moreover, the adults from diet C did not have the highest body lipid content. The profound discrepancy demonstrated that sodium-deficient individuals discarded larger quantities of waste products such as exuviae and meconium during metamorphosis. Further research will shed light on this issue.

## Conclusion

In present paper, we report a comparative analysis of the effects of dietary sodium on performance, flight and compensation strategies in *H. armigera*, a rarely mud-puddling insect species in nature. We found that sodium deficiency resulted in rapid growth and development of larvae, decreased larvae survival rate, and reduced flight speed of adults. *H. armigera *ingested a large quantity of larval food, increased larval cannibalism incidence and harvested sodium at adult stage to compensate for sodium deficiency.

## Competing interests

The authors declare that they have no competing interests.

## Authors' contributions

KX and KS carried out all tests. JFZ participated in the research and performed the statistical analysis. GQL proposed the research idea, designed research, wrote all versions of manuscripts. All authors have read and approved the final manuscript.
